# *Hura crepitans* L. Extract: Phytochemical Characterization, Antioxidant Activity, and Nanoformulation

**DOI:** 10.3390/pharmaceutics12060553

**Published:** 2020-06-15

**Authors:** Antonio Vassallo, Maria Francesca Armentano, Rocchina Miglionico, Carla Caddeo, Claudia Chirollo, Maria Josefina Gualtieri, Angela Ostuni, Faustino Bisaccia, Immacolata Faraone, Luigi Milella

**Affiliations:** 1 Department of Scienze, University of Basilicata, Viale dell’Ateneo Lucano 10, 85100 Potenza, Italy; antonio.vassallo@unibas.it (A.V.); mariafrancesca.armentano@unibas.it (M.F.A.); rocchina.miglionico@virgilio.it (R.M.); angela.ostuni@unibas.it (A.O.); faustino.bisaccia@unibas.it (F.B.); immacolata.faraone@unibas.it (I.F.); luigi.milella@unibas.it (L.M.); 2 Spinoff BioActiPlant s.r.l., Viale dell’Ateneo Lucano 10, 85100 Potenza, Italy; 3 Department of Scienze della Vita e dell’Ambiente, Sezione di Scienze del Farmaco, University of Cagliari, Via Ospedale 72, 09124 Cagliari, Italy; 4 Department of Veterinary Medicine and Animal Production, University of Napoli Federico II, Via Delpino 1, 80137 Napoli, Italy; claudia.chirollo@unina.it; 5 Department of Pharmacognosy and Organic Medicaments, University of Los Andes, 5101 Mérida, Venezuela; gualtier@ula.ve

**Keywords:** *Hura crepitans* L., extracts, liposomes, antioxidant activity, cytoprotective effect

## Abstract

The purpose of this study was to improve the knowledge on *Hura crepitans* L., a plant belonging to the Euphorbiaceae family that, on the one hand, is known to be toxic, but on the other, is a source of polyphenols with health-promoting effects. Different green extraction methods were applied, varying solvent, temperature, and duration of extraction, which can influence the phytochemical profile and biological activity of plant extracts, and the extracts were fully characterized. Aqueous extracts exhibited a superior antioxidant activity, as indicated by different spectrophotometric tests, and were cytoprotective to HepG2 cells used as model cells. Liquid chromatography–mass spectrometry analyses were performed to identify the secondary metabolites involved in these effects and demonstrated that solvent, duration, and temperature indeed influenced the extraction of polyphenols. Furthermore, the most promising extract, in terms of antioxidant potential, was incorporated into liposomes with the aim of promoting cell interaction and enhancing the antioxidant activity.

## 1. Introduction

*Hura crepitans* L. belongs to the Euphorbiaceae family. It is a tree growing up to 40 m high, characterized by dark, pointed (conical) spines. Its common name “Monkey-no-climb” refers to the characteristic spiny trunk. *H. crepitans* is known for many ethnomedicinal applications [1,2], but also for its toxicity [3]. Indeed, the latex is used as arrow poison and is said to cause ailing teeth to fall out. The milky sap is known to be a poison to fish, due to the presence of huratoxine and hexahydrohuratoxin, two lectins with hemagglutinating activity that inhibit protein synthesis [2,3]. Huratoxin was demonstrated to be more potent than callicarpone, isolated from *Callicarpa candicans* (Burm. f.) Hochr., and rotenone, a strong inhibitor of complex I of the mitochondrial respiratory chain [3,4,5]. On the other hand, *H. crepitans* leaves, stem bark, roots, and seeds have several therapeutic applications, which include the treatment of skin diseases, rheumatism, intestinal worms in leprosy [1,2]. A few studies reported the presence of flavonoids, phenolic acid, carotenoids, terpenes in root, stem bark, and leaf extracts of *H. crepitans*, especially in aqueous extracts [1]. These compounds are secondary metabolites involved in the defense of plants that play a key role in reducing oxidative stress, which is a prominent cause of various human diseases, such as cancer, neurodegenerative diseases, diabetes, and obesity [6]. Solvent, temperature, and duration of extraction can influence the phytochemical profile and biological activity of plant extracts [7,8]. Plant polyphenols are structurally heterogeneous, and their solubility depends on the chemical structure, which may vary from simple to highly polymerized compounds. Thus, the choice of extraction solvent is one of the most relevant steps in the extraction process. Generally, methanol, ethanol, propanol, acetone, ethyl acetate, and their mixture with water are used. According to the literature [7,8], mixtures of water and ethanol are more efficient in extracting total polyphenols than the corresponding mono-component solvent system. Moreover, the amount of water improves polyphenols yield [7].

Over the past years, extensive research has been devoted to polyphenols, highlighting their potential in therapy, which is ascribed to a wide range of biological activities, such as antioxidant, anti-inflammatory, antibacterial, and antiviral. However, polyphenols are characterized by poor solubility, low chemical stability, and low bioavailability, which limit their application in vivo [9,10]. The incorporation of plant polyphenols and extracts into micro/nanocarriers has been shown to increase their efficacy by protecting the active compounds from degradation, enhancing their solubility and bioavailability, and delivering adequate concentrations to the target site [11,12]. Therefore, this study aimed to increase scientific knowledge on the chemical composition of *H. crepitans* L. extracts, with a focus on the identification of the best extraction conditions to recover the highest amount of polyphenols and achieve the highest antioxidant activity. Moreover, the most promising extract was loaded into liposomes, and the enhancement of the antioxidant activity was investigated in cells.

## 2. Materials and Methods

### 2.1. Chemicals and Reagents

The following reagents were purchased from Sigma–Aldrich S.p.A. (Milan, Italy): sodium acetate trihydrate (CAS number 6131-90-4), 2,4,6-tripyridyl-*s*-triazine (TPTZ; CAS number 3682-35-7), iron (III) chloride hexahydrate (FeCl_3_ 6H_2_O; CAS number 10025-77-1), Folin–Ciocalteu reagent (MDL number MFCD00132625), 1,1-diphenyl-2-picryl hydrazyl radical (DPPH; CAS number 1898-66-4), β-carotene (CAS number 7235-40-7), linoleic acid (CAS number 60-33-3), Tween 20 (CAS number 9005-64-5), 6-hydroxy-2,5,7,8-tetramethylchroman-2-carboxylic acid (Trolox; CAS number 53188-07-1), gallic acid (CAS number 149-91-7), butylated hydroxytoluene (BHT, 2,6-bis(1,1-dimethylethyl)-4-methylphenol; CAS number 128-37-0), β-nicotinamide adenine dinucleotide reduced form (NADH; CAS Number 104809-32-7), phenazinemethosulfate (PMS; CAS number 299-11-6), nitrotetrazolium blue chloride (NBT; CAS number 298-83-9), 5,5′-dithio-bis(2-nitrobenzoic acid) (DTNB; CAS number 69-78-3), sodium nitroprusside dehydrate (SNP; CAS number 13755-38-9), *L*-ascorbic acid (CAS number 50-81-7), sulphanilamide (CAS number 63-74-1), Dulbecco’s Modified Eagle Medium (DMEM), dimethyl sulfoxide (DMSO; CAS number 67-68-5), 3-(4,5-dimethyl-2-thiazolyl)-2,5-diphenyl-2H-tetrazolium bromide dye (MTT; CAS number 298-93-1) and 2′,7′-dichlorodihydrofluorescein diacetate (DCFH-DA; CAS 4091-99-0).

Chloroform (CAS number 67-66-3), *n*-hexane (CAS number 110-54-3), glacial acetic acid (CAS number 64-19-7), and methanol (CAS number 67-56-1) were purchased from Carlo Erba (Milan, Italy). Solvents used for liquid chromatography–mass spectrometry analyses and extraction, as well as water (CAS number 7732-18-5), were purchased from VWR (Milan, Italy), while acetonitrile (CAS number 75-05-8) and formic acid (CAS number 64-18-6) were purchased from Merck (Darmstadt, Germany).

Trypsin-ethylenediaminetetraacetic acid solution (CAS number 9002-07-7), fetal bovine serum (FBS), glutamine (CAS number 56-85-9), penicillin-streptomycin, and phosphate buffer solution (PBS) were purchased from Euroclone (Milan, Italy). Soy lecithin (CAS number 8002-43-5) was purchased from Galeno (Carmignano, Prato, Italy).

### 2.2. Plant Material

*Hura crepitans* L. (HC) leaves were collected in Venezuela in 2018. A voucher specimen is stored at the Herbarium MERF, Faculty of Pharmacy and Bioanalysis at the University of Los Andes (Mérida, Venezuela), 1460 m above sea level (Voucher 001). The leaves were dried, powdered, and subjected to extraction by using different methods and solvents. A part of the dried leaves (1 g) was extracted with water (50 mL) by maceration, infusion, or decoction, obtaining three extracts: decoction (HC-D), infuse (HC-I), and macerate (HC-M), respectively. In more detail, the dried leaves were extracted by dynamic maceration at room temperature (25 °C) for 2 h; for the infusion, boiling water was added to dried leaves and kept in contact for 15 min; the decoction was obtained by boiling the dried leaves in water for 10 min. After the extraction, the three obtained solutions were kept in the dark, filtered with 17–25 µm cellulose filter, and dried by using a rotary evaporator.

Another part of the dried leaves (700 g) was extracted with 600 mL of *n*-hexane, chloroform, chloroform : methanol 9 : 1, or methanol by dynamic maceration at room temperature, obtaining four extracts: HC-H, HC-C, HC-CM, and HC-MeOH, respectively. The methanol extract was subjected to liquid/liquid repartitioning (R) using butanol or water, obtaining two additional extracts HC-R/BuOH and HC-R/H_2_O, respectively. All the extracts were dried and stored at 4 °C until use.

### 2.3. Total Polyphenolic Content (TPC)

To quantify the total polyphenolic content (TPC) of the dried extracts, the Folin–Ciocalteu assay was performed. Four hundred and twenty-five microliters of distilled water and 75 μL of gallic acid (reference) or extract were added to 500 μL of Folin–Ciocalteu reagent and 500 μL of Na_2_CO_3_ (10% *v/v* in H_2_O). The samples were vortexed and incubated for 1 h in the dark. After incubation, the absorbance was measured at 723 nm. All the reactions were performed in triplicate. Gallic acid was used to plot a standard curve. The results were expressed as mg of gallic acid equivalent (GAE)/g of dried extract [13,14]. All spectrophotometric measurements were performed by using the SPECTROstar^Nano^ (BMG Labtech, Ortenberg, Germany), if not otherwise specified.

### 2.4. DPPH Free Radical Scavenging Test

The free radical scavenging activity of the *H. crepitans* extracts was evaluated based on the scavenging of DPPH radical. Trolox was used as a standard. As described by Fidelis et al. [15], 50 μL of different dilutions of Trolox or extract was added to 200 μL of DPPH methanol solution (100 μM) in a 96-well plate. The absorbance was measured at 515 nm after 30 min of incubation in the dark at room temperature. A decrease in the absorbance of the DPPH solution indicates an increase in the radical scavenging activity of a sample [16]. The results were expressed as mg of Trolox equivalents (TE)/g of dried extract. Each reaction was performed in triplicate.

### 2.5. Ferric Reducing Antioxidant Power (FRAP)

The FRAP assay was performed to evaluate the reducing power of the *H. crepitans* extracts. Twenty-five microliters of Trolox (reference) or extract was added to 225 μL of FRAP reagent. The latter was composed of 300 mM acetate buffer (pH 3.6), 20 mM ferric chloride hexahydrate (FeCl_3_ 6H_2_O) in distilled water, and 10 mM TPTZ in 40 mM HCl, in a 10:1:1 ratio. The mixture was incubated at 37 °C for 40 min in the dark. The absorbance of the solution was measured at 593 nm. Each reaction was performed in triplicate. The results were expressed as mg of Trolox equivalents (TE)/g of dried extract [6].

### 2.6. β-Carotene Bleaching Test (BCB)

The β-carotene bleaching method (BCB) was used to evaluate the capacity of *H. crepitans* extracts to inhibit lipid peroxidation [6]. The β-carotene/linoleic acid emulsion (950 μL) was added to the extract or solvent as blank (50 μL). BHT was used as positive control. Two hundred and fifty microliters of this solution was transferred to a 96-well plate and incubated for 3 h at 50 °C. The absorbance was measured at 470 nm at 0, 30, 60, 90, 120, 150, and 180 min. The results were expressed as a percentage of β-carotene bleaching inhibition (%AA) and calculated according to Equation (1):

%AA = [1 − (A sample T_0′_ − A sample T_180′_) / (A blank T_0′_ − A blank T_180′_)] × 100 (1)

### 2.7. Superoxide Free Radical Scavenging Test

The ability of *H. crepitans* extracts to scavenge superoxide radical was evaluated spectrophotometrically according to a previously described procedure [17]. Superoxide radicals were generated by the phenazinemethosulfate-*β*-nicotinamide adenine dinucleotide (PMS-NADH) system. Several dilutions of the extracts (40 μL) or PBS as blank, NADH (40 μL), and NBT (130 μL) were placed in a 96-well plate. The reaction was started by adding PMS (40 μL) to the mixture and carried out for 2 min at room temperature. The absorbance was measured at 560 nm. For each extract, five different concentrations were tested. The results were expressed as the concentration inhibiting 50% of radical activity in mg/mL (IC_50_). Ascorbic acid was used as a positive control.

### 2.8. Nitric Oxide Radical Scavenging Activity

A nitric oxide radical (^•^NO) was generated *in vitro* from sodium nitroprussiate dehydrate (SNP) and measured by the Griess reaction [6]. SNP solution (80 μL, 6 mg/mL) was prepared in phosphate buffer (2% H_3_PO_4_, pH 7.4) and mixed with 90 μL of different concentrations of the extracts, in a 96-well plate. The mixture was incubated for 1 h at room temperature under light. Thereafter, 80 μL of Griess reagent (1:1 mixture (*v/v*) of 1% sulfanilamide and 0.1% *N*-(1-naphthyl) ethylenediamine in 2% H_3_PO_4_) were added, and the mixture was incubated for 10 min in the dark. The absorbance was measured at 560 nm. The results were expressed as IC_50_, and ascorbic acid was used as positive control.

### 2.9. LC-ESI/LTQOrbitrap/MS Analysis

To analyze the phytochemical profile of the aqueous *H. crepitans* extracts, an in-house High Performance Liquid Chromatography (HPLC) method coupled with a mass spectrometer, which associates the linear trap quadrupole and OrbiTrap mass analyzer, was used. LC-ESI/LTQOrbitrap/MS analyses were performed in positive and negative ion modes by using a Thermo Scientific Accela 600 HPLC system (coupled to an LTQ OrbiTrap XL mass spectrometer (Thermo Scientific, Bremen, Germany). Separation was achieved by using a Luna C_18_ column (2.5 μm; 100 × 2.10 mm; Phenomenex, Aschaffenburg, Germany). The mobile phases were water + 0.1% formic acid (solvent A) and acetonitrile (solvent B). The flow rate was 0.2 mL/min, and the gradient was the following: 2% of B at 0 min until 1 min, 40% until 21 min, 95% at 22 min, until 25 min, returning to 2% of B at 26 min until 35 min.

The MS setting was the following: in positive ion mode, source voltage 3 kV, capillary voltage 49 V, tube lens voltage 120 V; in negative ion mode, source voltage 5 kV, capillary voltage −48 V, tube lens voltage −176.47 V. Capillary temperature for both positive and negative ion modes was 280 °C. MS spectra were acquired by full range acquisition covering *m/z* 150–1000.

Data were acquired by using Xcalibur software version 2.1, and for fragmentation studies, a data-dependent scan experiment was carried out by selecting precursor ions as the most intensive peak in LC-MS analysis. Identification of compounds was based on retention times, accurate mass measurements, MS/MS data, exploration of specific spectral libraries and public repositories for MS-based metabolomic analysis [18], and comparison with data reported in the literature [19,20,21].

### 2.10. Liposome Preparation and Characterization

For the preparation of liposomes, 150 mg/mL of soy lecithin and 5 mg/mL of HC-M extract were weighed in a glass vial, dispersed in water and sonicated (20 cycles, 5 s on and 2 s off; 13 μm of probe amplitude) with a high-intensity ultrasonic disintegrator (Soniprep 150, MSE Crowley, London, UK) [16,21].

The average diameter and polydispersity index (PI; a measure of the size distribution width) of the vesicles were determined by dynamic light scattering using a Zetasizer nano-ZS (Malvern Instruments, Worcestershire, UK). Zeta potential was estimated using the Zetasizer nano-ZS by means of the M3-PALS (mixed mode measurement-phase analysis light scattering) technique, which measures the particle electrophoretic mobility. The samples (*n* > 10) were diluted with water (1:100) and analyzed at 25 °C. For comparative purposes, empty liposomes (i.e., without extract) were also prepared and characterized.

### 2.11. Cell Culture and Treatment with Extracts

Human hepatoma cells (HepG2) were cultured in DMEM supplemented with 10% FBS, penicillin (100 units/mL), and streptomycin (100 units/mL) in a humidified 5% CO_2_ incubator at 37 °C. All the tested extracts were dissolved in DMSO and diluted to the required concentrations with DMEM. The final DMSO concentration in cell cultures was never greater than 0.8%, which has no effect on cell viability. DMSO-treated cells were used as controls in all the experiments. HC-M extract loaded liposomes (LHC-M) were diluted to the required concentrations with DMEM. The cells were treated at 60–70% confluence, at passages 4 to 10.

### 2.12. Cell Viability Assay

Cell viability was evaluated by a colorimetric assay based on the conversion of the yellow tetrazolium salt MTT into purple insoluble formazan by succinate dehydrogenase enzyme of viable cells. HepG2 cells were seeded in a 96-well plate (10^4^ cells/well), incubated overnight and treated, for 24 and 48 h, with different concentrations of *H. crepitans* extracts (50, 100, 200, 300, 400 µg/mL for HC-MeOH and HC-R/BuOH extracts, and 200, 300, 400, 600, 800 µg/mL for HC-D, HC-I, and HC-M extracts), and liposomes (LHC-M; 3.125, 6.25, 12.5, 25, 50 µg/mL) for 24 h. After medium removal, the cells were washed with PBS and incubated for 4 h with 0.75 mg/mL of MTT solution in PBS. Then, the solution was removed, and the cells were lysed using a solubilization mixture (1:1 DMSO:isopropanol). The solubilized formazan product was spectrophotometrically quantified at 560 nm using a Multiskan Go microplate spectrophotometer (Thermo Scientific, Bremen, Germany).

### 2.13. Measurement of Intracellular ROS

The intracellular reactive oxygen species (ROS) level was measured with 2′,7′-dichlorodihydrofluorescein diacetate (DCFH-DA) as previously described [22]. Briefly, HepG2 cells were plated at a density of 2 × 10^5^ cells/well in a 24-well plate, pre-treated with different concentrations of HC-D, HC-I, and HC-M extracts (200, 400, and 600 µg/mL) or liposomes (LHC-M; 3.125, 6.25, 12.5 µg/mL) for 24 h, and then incubated for 1 h with 2 mM H_2_O_2_. Finally, the cells were stained with 10 µM DCFH-DA for 30 min at 37 °C in the dark, and fluorescence was measured by BD FACSCanto II (BD Pharmingen, San Jose, CA, US) at an excitation wavelength of 485 nm and an emission wavelength of 515–540 nm.

### 2.14. Statistical Analysis

Data are expressed as means ± standard deviations (SD). Statistical analysis was performed by using GraphPad Prism 7 Software, Inc. (San Diego, CA, US). One-way ANOVA test or two-way ANOVA test were performed, followed by Tukey–Kramer or Dunnett’s post-hoc tests. A difference was considered significant when *p* < 0.05.

## 3. Results

### 3.1. Total Polyphenols Content and Antioxidant Activity

The total polyphenol content of *H. crepitans* extracts was analyzed. The aqueous extracts obtained by decoction (HC-D; 317.5 mg GAE/g of extract), infusion (HC-I; 308.5 mg GAE/g of extract), and maceration (HC-M; 257.4 mg GAE/g of extract) showed the higher content of polyphenols, followed by the methanol extract (HC-MeOH; 194.6 mg GAE/g of extract) and butanol–methanol extract (HC-R/BuOH; 164.6 mg GAE/g of extract) (Table 1). The solvent and the extraction technique are known to influence the metabolite profile and antioxidant activity of an extract. Polar solvents are frequently employed for the recovery of polyphenols from plant material. Water or aqueous mixtures containing methanol, ethanol, acetone, and ethyl acetate are the most suitable polar solvents [23].

Three different assays were used to evaluate the antioxidant activity of the extracts (Table 1). The radical scavenging capacity was determined by DPPH, SO, and NO assays. HC-I, HC-R/BuOH, and HC-MeOH showed the greatest radical scavenging activity using the DPPH test (1229.3, 1019.1, and 1110.3 mg TE/g, respectively). Most of the extracts showed good antioxidant activity against SO and NO physiological radicals. The results were expressed as IC_50_ in mg/mL; the values obtained for *H. crepitans* extracts were lower than that of ascorbic acid used as a standard (IC_50_: 0.24 mg/mL in SO assay and 3.04 mg/mL in NO assay). In particular, HC-I, HC-M, HC-D showed the greatest radical scavenging activity against SO and NO (Table 1).

The ability of the extracts to reduce ferric ions was studied with the FRAP test. The results showed that HC-I had the highest reducing power (7573.3 mg TE/g) (Table 1).

The antioxidant effect of the extracts on the peroxidation of linoleic acid in the β-carotene/linoleic acid system was investigated by means of the BCB test. The oxidation of linoleic acid generates peroxyl free radicals, which in turn oxidize the highly unsaturated β-carotene. The presence of antioxidants minimizes the oxidation of β-carotene. HC-MeOH (69.1%) and HC-R/BuOH (67.4%) showed the highest β-carotene bleaching inhibitory activity (Table 1).

**Figure 1:**
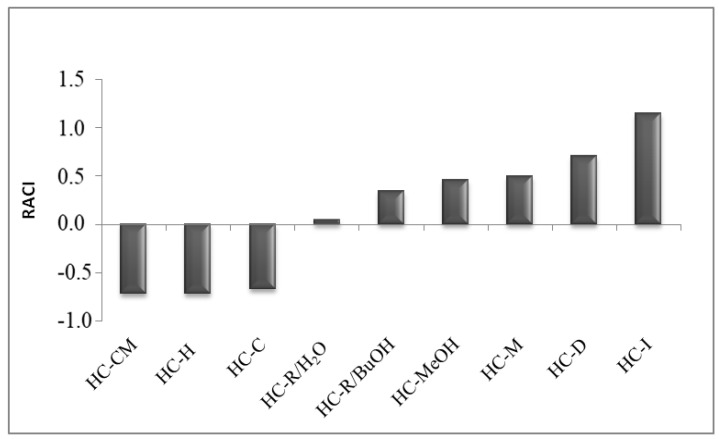
RACI (relative antioxidant capacity index) values obtained by comparing TPC, DPPH, FRAP, BCB, NO, and SO results. TPC: total polyphenolic content; DPPH: DPPH: 2,2-diphenyl-1-picrylhydrazyl; FRAP: ferric reducing antioxidant power; BCB: β-carotene bleaching assay; NO: nitric oxide; SO: superoxide anion.

The relative antioxidant capacity index (RACI) was calculated for all the tested extracts (Figure 1). RACI is an adimensional index that has been demonstrated to be a useful tool for comparing results from different antioxidant assays [6]. All the assays used to determine the antioxidant activity of the *H. crepitans* extracts were included in RACI calculation by using Excel software (Microsoft, Washington, US). The total content of polyphenols was also included because the principle of the test involves an electron-transfer reaction between phenolic compounds (or other reductants) and molybdenum under alkaline conditions, resulting in the formation of blue complexes that can be detected spectrophotometrically at 723 nm. Thus, the method was recently proposed for the determination of the total reducing capacity of samples, which reflects the cumulative capacity of both phenolic and non-phenolic compounds to reduce the Folin–Ciocalteu reagent [13]. According to the above-mentioned results of the antioxidant assays, the aqueous extracts were the most effective, with HC-I having the highest RACI (1.15). This is likely to be due to the optimal recovery of phenolic compounds provided by the high temperature used for the infusion (∼100 °C) in comparison with maceration (25 °C), and to the reduction in hydrolyzation or oxidation processes that might occur during decoction.

### 3.2. Cytotoxicity of H. crepitans Extracts

**Figure 2:**
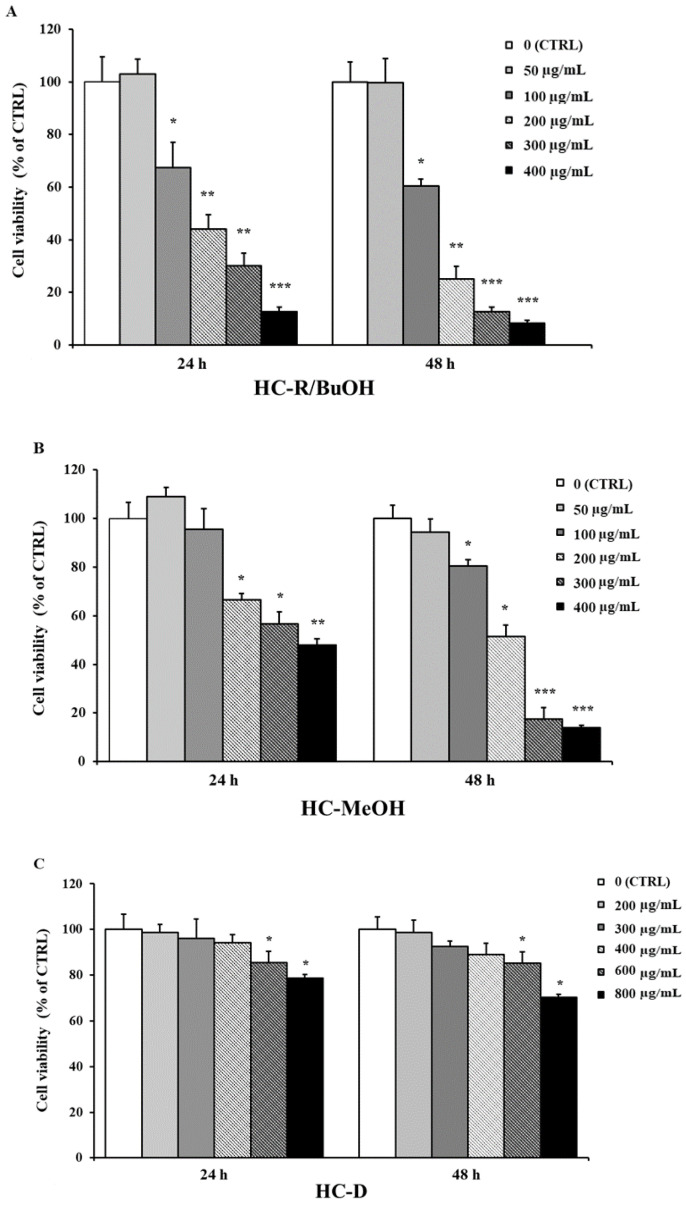

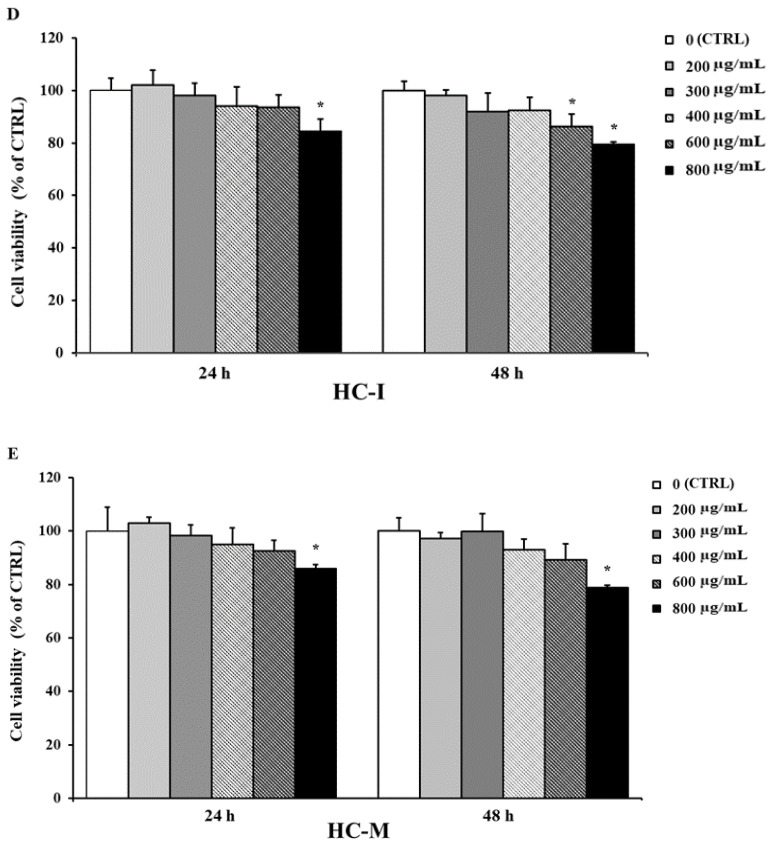
Viability of HepG2 cells treated for 24 and 48 h with different concentrations of (**A**) HC-R/BuOH, (**B**) HC-MeOH, (**C**) HC-D, (**D**) HC-I, (**E**) HC-M. Data are expressed as the mean ± SE of three independent experiments (*n* = 3) and were analyzed by one-way ANOVA followed by Dunnett’s post-hoc test, * *p* < 0.05, ** *p* < 0.01 and *** *p* < 0.001 vs. CTRL (100% viability).

HC-I, HC-M, HC-D, HC-MeOH, and HC-R/BuOH extracts displayed the greatest antioxidant activity in vitro, hence they were tested on cells. As shown in Figure 2A,B, HC-R/BuOH and HC-MeOH extracts exhibited a dose-dependent cytotoxicity on HepG2 cells, while the aqueous extracts (HC-D, HC-I, and HC-M; Figure 2C–E) showed no cytotoxicity, with 50% cell growth inhibition (IC_50_) values always greater than 800 µg/mL. Therefore, these three extracts were analyzed for phytochemical composition and tested for antioxidant activity in cells.

### 3.3. LC-ESI/LTQOrbitrap/MS

Given that HC-D, HC-I, HC-M aqueous extracts displayed great antioxidant activity in vitro and no cytotoxicity, they were characterized by LC-ESI/LTQOrbitrap/MS to identify the main components. The metabolites identified in *H. crepitans* extracts are reported in Table 2. LC–MS revealed the presence of 14 compounds identified as caffeic acid, gallic acid, 3,4-dihydroxybenzoic acid, syringic acid, epigallocatechin, rutin, isoquercetin, quercetin, myricetin, epicatechin, naringin, luteolin, resveratrol, and ferulic acid by comparing their *m/z* values in the total ion current (TIC) with those described in the literature [18,19,20,21]. Table 3 reports the content of phenolic compounds (mg/kg) of the aqueous extracts. The most abundant metabolites identified in all *H. crepitans* aqueous extracts were the phenols caffeic and gallic acid, the flavonols rutin and quercetin, and the flavanone naringin. In particular, caffeic acid, gallic acid, and quercetin were much more abundant in HC-M extract than the other extracts, while rutin and naringin were present in amounts similar to those found in HC-D and HC-I extracts, respectively.

### 3.4. Protective Effect of H. crepitans Extracts against ROS Intracellular Production

**Figure 3:**
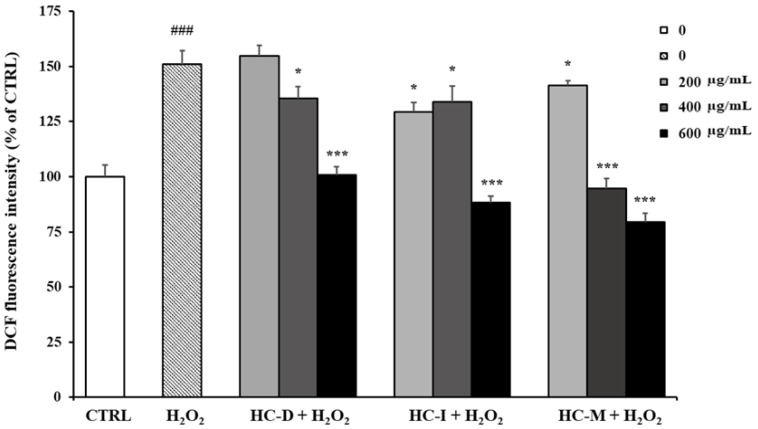
Effect of *H. crepitans* extracts on H_2_O_2_-induced intracellular ROS generation in HepG2 cells. The cells were pre-treated with the extracts at different concentrations (200, 400, and 600 μg/mL) for 24 h and subsequently incubated for 1 h with 2 mM H_2_O_2_. ROS generation was measured by flow cytometry using DCFH-DA staining. Data are expressed as the mean ± SE of three independent experiments (n = 3) and were analyzed by one-way ANOVA followed by Dunnett’s post-hoc test. ^###^ *p* < 0.001 vs. CTRL, * *p* < 0.05 and *** *p* < 0.001 vs. H_2_O_2_-treated cells.

The antioxidant activity of the *H. crepitans* aqueous extracts was also evaluated in terms of anti-ROS activity in cells. The measurement of ROS gives an indication of the level of oxidative stress. H_2_O_2_ was used as the source of ROS. Indeed, in cells, H_2_O_2_ is converted to hydroxyl radicals and causes oxidation of dichlorodihydrofluorescein (DCFH) to dichlorofluorescein (DCF) complex, a fluorescent compound. After H_2_O_2_-induced oxidative stress, pre-treated HepG2 cells with a higher concentration of *H. crepitans* aqueous extracts (HC-D, HC-I, HC-M; 600 μg/mL) gave lower fluorescence values as compared to cells treated with H_2_O_2_ only (Figure 3). The lower concentration of the extracts (200 μg/mL) displayed an effect similar or slightly different from the stressed cells. Of note, HC-M showed a significant antioxidant activity already at 400 μg/mL, unlike HC-D and HC-I, which justifies the choice of using this extract for the liposomal formulation.

### 3.5. HC-M Extract Liposomal Formulation: Characterization and Bioactivity in Cells

Liposomes were prepared by a simple method involving the sonication of the phospholipid (soy lecithin) and HC-M extract in water. To evaluate the effect of the incorporation of the extract on the vesicle arrangement, empty liposomes were also prepared and characterized. As shown in Table 4, empty liposomes displayed small size (73 nm), good homogeneity (P.I. 0.25), and highly negative zeta potential (∼−50 mV). When the HC-M extract was incorporated, there was a slight increase in size (84 nm) with an improvement in the homogeneity (P.I. 0.20). This can be explained by an arrangement of the extract with the phospholipid during the liposome formation, which alters the geometric packing of the bilayer and thus the vesicle diameter. On the other hand, the effect of the extract on the vesicle surface charge was negligible.

**Figure 4:**
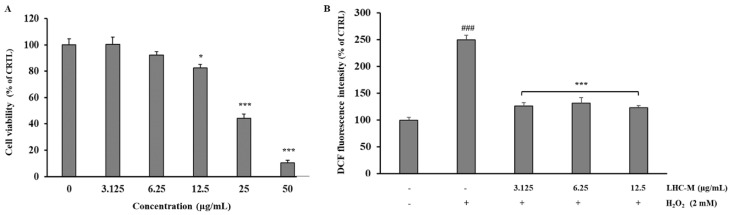
Effect of *H. crepitans* extract loaded liposomes (LHC-M) on cell viability and H_2_O_2_-induced intracellular ROS generation in HepG2 cells. (**A**) Viability of HepG2 cells treated for 24 h with different concentrations of LHC-M. Untreated cells were used as control (CTRL; 100% viability). Data are expressed as the mean ± SE of three independent experiments (n = 3) and were analyzed by one-way ANOVA followed by Dunnett’s post hoc test, * *p* < 0.05 and *** *p* < 0.001 vs. CTRL. (**B**) Cells were pre-treated for 24 h with LHC-M at different concentrations (3.125, 6.25 and 12.5 μg/mL) and subsequently incubated for 1 h with 2 mM H_2_O_2_. ROS generation was measured by flow cytometry using 2′,7′-dichlorodihydrofluorescein diacetate (DCFH-DA) staining. Data are expressed as the mean ± SE of three independent experiments (n = 3) and were analyzed by one-way ANOVA followed by Dunnett’s post hoc test. ^###^ *p* < 0.001 vs. CTRL (HepG2 cells treated with vehicle), *** *p* < 0.001 vs. H_2_O_2_-treated cells.

Both the MTT test and ROS measurements were performed on HepG2 cells treated with HC-M extract loaded liposomes (LHC-M). As shown in Figure 4A, a dose-dependent cytotoxicity was observed: the IC_50_ value was approximately 30 μg/mL. Based on these results, concentrations lower than the IC_50_ value (3.125, 6.25, 12.5 µg/mL) were used to assess the antioxidant activity of the liposomal formulation of HC-M extract. Figure 4B shows that the liposomes were able to maintain ROS levels close to endogenous ones, preventing ROS production already at the lower concentration (3.125 µg/mL), without statistical difference between the tested concentrations.

## 4. Discussion

The antioxidant activity of different extracts of *H. crepitans* leaves was evaluated by means of six different in vitro tests. Solvent and temperature of extraction are known to influence the polyphenol content and biological activity of extracts [7]. The aqueous extracts obtained by decoction, infusion, and maceration (HC-D, HC-I, HC-M) showed higher antioxidant activity, as compared to the less polar extracts (Figure 1). Greater activity of the aqueous extracts was also observed in cells. The aqueous extracts showed no cytotoxicity, unlike HC-R/BuOH and HC-MeOH extracts, which reduced cell viability in a dose-dependent manner (Figure 2). This is reasonably due to the solvents used for the extraction process. Besides the biocompatibility, the aqueous extracts showed a strong ability to counteract free radicals (ROS). Indeed, HC-D, HC-I, and HC-M extracts (at 400 and mostly at 600 μg/mL) protected the cells from oxidative stress, keeping the intracellular ROS levels equal to the control (Figure 3).

The different behavior of the extracts in cells is probably due to the difference in composition. Previous studies reported the presence of huratoxin, a diterpene with piscicidal activity, in ether and methanol extracts of *H. crepitans* [3]. On the contrary, huratoxin was not detected by GC-MS in aqueous extracts of *H. crepitans* leaves [1]. In this work, LC-MS was performed to identify the secondary metabolites, particularly polyphenols with antioxidant activity, in the *H. crepitans* aqueous extracts. In agreement with the literature [24], the aqueous extracts contained several phenolic compounds (Table 3). Moreover, two new compounds were identified: syringic acid and caffeic acid (Table 3). These metabolites have shown *in vitro* and *in vivo* antioxidant activity, reducing the levels of the free radicals and promoting the expression of important antioxidant enzymes, such as glutathione and catalase [21,25]. As previously reported, temperature and duration of extraction influence the phytochemical profile of *H. crepitans* leaf extracts [8,9]. In this work, HC-M extract, which was obtained at 25 °C, showed a greater concentration of phenolic compounds not detected in the extracts obtained at high temperature (∼100 °C) (i.e., HC-D and HC-I) [9]. Indeed, the HC-M extract contained twice the amount of caffeic acid (10650.4 mg kg^−1^), gallic acid (47384.0 mg kg^−1^), myricetin (428.3 mg kg^−1^), and luteolin (119.6 mg kg^−1^) found in HC-D and HC-I extracts (Table 3). These results might explain the potent anti-ROS activity of HC-M extract already at 400 μg/mL (Figure 3). Based on these findings, HC-M extract was formulated in liposomes and tested in cells. Interestingly, a quite low concentration (∼3 μg/mL) was needed to prevent the oxidative stress caused by ROS, as compared to 400 μg/mL required for the free extract. This confirms the striking advantage provided by liposomes, which are known to facilitate the interaction with cells and allow the release of the payload in the cytoplasm, where the antioxidant activity is exerted.

## 5. Conclusions

The use of different extraction techniques and solvents allowed the production of different types of extracts. We found that solvent, temperature, and duration of extraction influenced the phytochemical profile and biological activity of *H. crepitans* leaf extracts. The results suggest the use of *H. crepitans*, traditionally known for its toxicity, as a source of health-promoting compounds. Indeed, aqueous extracts demonstrated a good antioxidant activity, especially when incorporated into liposomes, which supports the use of these herbal formulations for the treatment of pathologies caused by oxidative stress. Moreover, the cytotoxic effect of the butanol and methanol extracts on HepG2 cells will be further investigated, as they could represent a new anti-cancer strategy.

## Figures and Tables

**Table 1:** Antioxidant activity of *H. crepitans* leaf extracts.

Extract	Solvent	Extraction Temp	TPC ^§^	DPPH ^§§^	SO ^§§§^	NO ^§§§^	FRAP ^§§^	BCB ^§§§§^
HC-D	H_2_O	≥ 100 °C	317.5 ± 4.2 ^a^	759.9 ± 46.4 ^b^	0.02 ± 0.005 ^a^	0.22 ± 0.02 ^a^	824.1 ± 74.2 ^b^	48.5 ± 5.5 ^a^
HC-I	H_2_O	≤100 °C	308.5 ± 8.1 ^a^	1229.3 ± 9.3 ^a^	0.02 ± 0.001 ^a^	0.35 ± 0.08 ^a,b^	7573.3 ± 656.3 ^a^	49.1 ± 7.7 ^a^
HC-M	H_2_O	≅ 20 °C	257.4 ± 5.6 ^b^	478.8 ± 3.9 ^c^	0.02 ± 0.001 ^a^	0.25 ± 0.08 ^a^	754.3 ± 76.9 ^b,c^	48.1 ± 1.6 ^a^
HC-H	*n*-Hex	≅ 20 °C	46.4 ± 0.8 ^c^	998.1 ± 15.8 ^d^	nc	nc	538.5 ± 67.7 ^b,c^	nc
HC-MeOH	MeOH	≅ 20 °C	194.6 ± 3.0 ^d^	1110.3 ± 44.1 ^e^	0.07 ± 0.008 ^b^	0.66 ± 0.26 ^b^	3645.9 ± 436.8 ^d^	69.1 ± 4.1 ^a^
HC-R/H_2_O	MeOH R/H_2_O	≅ 20 °C	90.8 ± 4.9 ^e^	332.7 ± 26.3 ^f^	0.12 ± 0.01 ^c^	0.21 ± 0.16 ^a^	1795.2 ± 191.6 ^e^	54.1 ± 5.8 ^a^
HC-R/BuOH	MeOH R/BuOH	≅ 20 °C	164.6 ± 15.4 ^f^	1019.1 ± 13.3 ^d^	0.06 ± 0.008 ^b^	1.12 ± 0.23 ^c^	3801.6 ± 507.7 ^d^	67.4 ± 5.8 ^a^
HC-C	CHCl_3_	≅ 20 °C	62.4 ± 0.9 ^c^	nc	nc-	nc	230.0 ± 33.1 ^b,c^	55.4 ± 3.6 ^a^
HC-CM	CHCl_3_-MeOH	≅ 20 °C	40.9 ± 6.6 ^c^	nc	nc-	nc	333.0 ± 42.6 ^b,c^	52.9 ± 2.9 ^a^

Values are expressed as the means ± SD of three replicates from three independent experiments in **^§^** mg of gallic acid equivalents/g of extract; **^§§^** mg of Trolox equivalents/g of extract; **^§§§^** concentration required for 50% inhibition in mg/mL; **^§§§§^** antioxidant activity% at an initial concentration of 2.5 mg/mL. nc = not calculable at the tested concentrations. Two-way ANOVA followed by Tukey–Kramer post-hoc analysis was used, and differences were considered significant when *p* < 0.05 and are indicated with superscripts letters: for values with a different letter, the difference is statistically significant (*p* < 0.05). Extracts were obtained by decoction (HC-D), infusion (HC-I), and maceration (HC-M), or by using *n*-hexane (HC-H), chloroform (HC-C), chloroform : methanol 9 : 1 (HC-CM), methanol (HC-MeOH) solvents, or by liquid/liquid extraction of HC-MeOH with butanol or water (HC-R/BuOH and HC-R/H_2_O). TPC: total polyphenolic content; DPPH: 2,2-diphenyl-1-picrylhydrazyl; SO: superoxide anion; NO: nitric oxide radical; FRAP: ferric reducing antioxidant power; BCB: β-carotene bleaching assay.

**Table 2:** Metabolites identified in *H. crepitans* aqueous extracts by Liquid chromatography–mass spectrometry.

n	Rt (Min)	Molecular Formula	[M-H]^−^	MS/MS	Identity
1	1.3	C_9_H_8_O_4_	179.0559	161; 151; 135; 133; 117; 97	Caffeic acid
2	5.6	C_7_H_6_O_5_	169.0301	151; 141; 125; 83	Gallic acid
3	8.2	C_7_H_6_O_4_	152.9871	135; 125; 119; 109; 97; 77	3,4-dihydroxybenzoic acid
4	14.7	C_9_H_10_O_5_	197.0964	n.s.	Syringic acid
5	15.0	C_15_H_14_O_7_	305.1924	288; 261	Epigallocatechin
6	15.3	C_27_H_30_O_16_	609.1616	271; 255; 179	Rutin
7	15.9	C_21_H_20_O_12_	463.0917	461; 301	Isoquercetin
8	16.2	C_15_H_10_O_7_	301.1122	273; 257; 245; 229; 213; 201; 185; 179; 151	Quercetin
9	16.3	C_15_H_10_O_8_	317.1703	n.s.	Myricetin
10	16.7	C_15_H_14_O_6_	288.976	245; 205; 203; 123; 109	Epicatechin
11	17.0	C_27_H_32_O_14_	579.2358	417; 399; 339; 301; 255; 227; 217; 179	Naringin
12	20.3	C_15_H_10_O_6_	285.0398	257; 241; 217; 199; 175; 151; 133	Luteolin
13	24.1	C_14_H_12_O_3_	227.2173	183; 181; 159; 143; 115	Resveratrol
14	24.5	C_10_H_10_O_4_	193.1645	n.s.	Ferulic acid

n.s.: no signal.

**Table 3:** Phenolic compounds (mg/kg) in *H. crepitans* extracts. Data represent the mean values ± SD from two separate experiments, each performed in triplicate. One-way ANOVA followed by Tukey–Kramer post-hoc analysis was used, and differences were considered significant when *p* < 0.05 and are indicated with different superscripts letters: for values with a different letter, the difference is statistically significant (*p* < 0.05).

Compound	HC-D	HC-I	HC-M
Caffeic acid	4850.0 ± 131.3 ^a^	5934.0 ± 158.4 ^b^	10650.4 ± 256.3 ^c^
Gallic acid	20056.0 ± 491.4 ^a^	14855.0 ± 333.4 ^b^	47384.0 ± 644.6 ^c^
3,4-dihydroxybenzoic acid	3943.0 ± 92.6 ^a^	3244.0 ± 72.1 ^b^	3613.0 ± 82.3 ^c^
Syringic acid	969.8 ± 21.2 ^a^	443.0 ± 10.1 ^b^	845.0 ± 18.1 ^c^
Epigallocatechin	523.5 ± 12.1 ^a^	357.2 ± 8.2 ^b^	356.0 ± 8.3 ^b^
Rutin	47197.6 ± 880.0 ^a^	39710.5 ± 782.8 ^b^	44678.0 ± 827.0 ^a^
Isoquercetin	602.1 ± 13.1 ^a^	377.6 ± 7.4 ^b^	419.1 ± 8.5 ^c^
Quercetin	9069.8 ± 216.7 ^a^	5046.6 ± 117.2 ^b^	11130.8 ± 254.3 ^c^
Myricetin	266.9 ± 5.7 ^a^	225.6 ± 4.6 ^a^	428.3 ± 8.7 ^b^
Epicatechin	668.2 ± 14.7 ^a^	431.2 ± 9.8 ^b^	388.2 ± 8.7 ^c^
Naringin	6425.5 ± 152.6 ^a^	8454.6 ± 181.4 ^b^	8250.0 ± 176.3 ^b^
Luteolin	46.9 ± 1.2 ^a^	46.4 ± 1.1 ^a^	119.6 ± 1.9 ^b^
Resveratrol	740.9 ± 13.5 ^a^	2113.3 ± 42.8 ^b^	697.1 ± 12.4 ^a^
Ferulic acid	TRACE	TRACE	TRACE

TRACE: minimum amount.

**Table 4:** Characteristics of empty liposomes and *H. crepitans* extract (HC-M) loaded liposomes: intensity-weighted mean hydrodynamic diameter, polydispersity index (P.I.), and zeta potential (ZP). Each value represents the mean ± SD, *n* > 10; ^#^ SD for P.I. values was always <0.03.

Formulation	Mean Diameter (nm ± SD)	P.I. ^#^	ZP (mV ± SD)
Empty liposomes	73 ± 7.8	0.25	−54 ± 6.6
HC-M liposomes	84 ± 7.6	0.20	−46 ± 8.0
